# Some Fallacies Concerning Syphilis

**Published:** 1890-06

**Authors:** 


Some Fallacies Concerning Syphilis. By E. L. Keyes, M. D. Geo. S. Davis, Detroit. 1890. Price, 25 cents.

Dr. Keyes, in this monograph, deals with thirteen popular fallacies in the pathology and treatment of syphilis, and clearly and concisely sets forth his views on the subject. The little book may be read in an hour, but it contains much very valuable information, especially to general practitioners who have not kept abreast with the literature of the subject.

